# Deliberate chemical attack: revisiting the lessons of the Tokyo subway attack

**DOI:** 10.1186/1757-7241-22-S1-A8

**Published:** 2014-07-07

**Authors:** Mark Byers

**Affiliations:** 1 London’s Air Ambulance, Royal London Hospital, London E1 1BB, UK

## 

The Japanese Sect Aum Shrinryko launched their terrorist attack on the Tokyo Underground on the 20 March 1995. Five terrorists boarded underground trains heading for the Kasumigaseki station carrying at least two bags each of liquid Sarin (Isopropyl methylphophonofluoridate ) wrapped in newspaper. These were punctured with the sharpened tip of an umbrella prior to the escape of the perpetrators and the liquid inside was allowed to run out and vaporise.

Sarin is the most volatile nerve agent and acts through anticholinesterase inhibitor. As acetylcholine is the transmitter at all neuromuscular junctions and throughout the central and peripheral nervous system, it has profound effects.

Sarin attaches to acetylcholinesterase preventing breakdown of acetylcholine. Muscarinic receptor hyperstimulation leads to constriction of all smooth muscles in the body, particularly manifest in bronchospasm and bronchorrhoea. Nicotinic simulation leads to voluntary muscle overactivation with muscle fasciculation and then muscle weakness. Central effects include coma, seizures and loss of central respiratory drive.

In total the attack led to 12 deaths, 54 severely injured, 980 people moderately affected and over 5000 people who sought medical assistance. Of note, 10% of ambulance staff and 110 (23 %) hospital staff were also affected. The numbers killed and injured reflects the fact that, even though the nerve agent was released during one of the busiest periods of transport activity, rush hour, the sarin was only 35% pure and only ten 500ml bags were used. They had the technology to do better – Tsuchiya (the Aum Chemist) had proved he could manufacture higher quality nerve agent, having produced 3kg of 90% pure Sarin from suitable precursors in only two months.

The Japanese published several papers on their experiences and common themes run through the lessons they identified. These included the need for physicians in command and control centres, the requirement for readily available personal protective equipment. They highlighted the difficulty of diagnosis and detection of the agent released and the problems of accessing appropriate antidotes. They also discussed the problems of decontamination procedures and the difficulties of crowd control [1].

Many of the lessons drawn by the Japanese remain valid today, but some have been forgotten, ignored or overtaken by events. Looking back with the benefit of hindsight and the passage of time these lessons need to be reviewed or re-iterated.

These include:

Communications will probably fail and rather than add layer upon layer of complexity and technology to overcome this problem, it may be better to accept communications will fail and put simpler strategies in place to manage this almost inevitable situation. Almost every major incident report mentions communication difficulties and failures and the solution seems to be more, not less technology [2]. This should be reviewed.

The early diagnosis of chemical exposure and initial management of the most serious casualties is best performed by highly trained and experienced clinical personnel using well understood toxidromes, aided by, but not substituted for, sensors and detectors. Given the complexities of this diagnosis, this task should be performed by a pre-hospital physician who can rapidly assimilate the complex presentation of undifferentiated disease and deliver a working hypothesis and treatment regime at the point of exposure.

Crowds will be difficult to control and will not remain at the scene to process through a bespoke decontamination facility. Be prepared for casualties to flee the scene and self-present at health care facilities. In a chemical terrorist incident all healthcare facilities should expect to receive casualties who self-refer and know how to deal with them. This requires education and practice.

The Emergency Services will not be able to adequately decontaminate all people involved in the incident in either a timely or meaningful way. Strategies are needed to stream casualties so that only those who will benefit from active decontamination undergo the procedure. In most mass exposure events, it is likely that the majority of those present will need either no decontamination or decontamination limited to undressing. More research into decontamination procedures is required to identify which patients need supervised decontamination, which need only to undress and which need no decontamination at all.

Central control will not be effective in a single large, or multiple small incidents. Resources and authority must be delegated to scene commanders in order to ensure the “man on the ground” has the necessary means to manage the appropriate area of authority. That is not to say that the Centre must not control the logistic reserve or retain control of casualty flow, but the delegation of responsibility to the lowest level is well recognized as essential by military commanders who recognize the difficulties of micromanaging complex situations [3].

Antidotes need to be immediately available. Holding antidotes in strategic national stockpiles will not work when an antidote is required in minutes. High concentration nerve agent vapour exposure will rapidly lead to death without immediate treatment, similarly the cyanides. Even the slower acting agents require treatment outside the timelines imposed by holding antidotes centrally in “strategic” stockpiles.

Nineteen years after the Tokyo Subway Sarin attack (and only a year after the use of nerve agents in Syria) we still have a great deal to learn about how to manage these incidents. Whilst this paper offers no answers it will have succeeded if it reignites the debate about how to manage these incidents.
